# The Elephant in the Room: The Need for Increased Integrative Therapies in Conventional Medical Settings

**DOI:** 10.3390/children5110154

**Published:** 2018-11-16

**Authors:** Missy Hall, Susanne M. Bifano, Leigh Leibel, Linda S. Golding, Shiu-Lin Tsai

**Affiliations:** 1 Child Life Department, NewYork-Presbyterian Morgan Stanley Children’s Hospital Columbia University Medical Center, 3959 Broadway, New York, NY 10032, USA; sub9053@nyp.org; 2 Division of Hematology/Oncology, Columbia University Medical Center, 161 Ft. Washington Ave, Suite 922, New York, NY 10032-3789, USA; LL3125@cumc.columbia.edu; 3 Pastoral Care, NewYork-Presbyterian Milstein Columbia University Medical Center, 622 W. 168th St., New York, NY 10032-3789, USA; lig9048@nyp.org; 4 Division of Pediatric Emergency Medicine, Department of Emergency Medicine, Columbia University College of Physicians and Surgeons, 3959 Broadway, CHN-W116, New York, NY 10032; st166@cumc.columbia.edu

**Keywords:** Integrative therapies, art therapy, music therapy, yoga therapy, acupuncture, pastoral care, creative arts therapy, pediatrics

## Abstract

Pediatric integrative therapy programs are essential to the treatment and well-being of patients. Identifying an effective integrative therapy model within conventional pediatric medical settings, however, often proves difficult. Our goal in this article is to explore varied solutions to increase access and inclusion of integrative therapies in an effort to promote best practice and holistic care. The main methods applied in this article are vignettes that illustrate how the integrative therapies in a metropolitan academic hospital successfully treat the patient by complementing conventional medicine. This leads to comprehensive care. The central finding of the article proposes viable solutions to increase interdisciplinary collaboration both internally within the institution and externally. Integrative therapists detail how they were able to increase visibility and yield best practice through increased educational initiatives and interdisciplinary collaboration.

## 1. Introduction

*And so these men of Indostan, disputed loud and long,*

*each in his own opinion, exceeding stiff and strong,*

*Though each was partly in the right, and all were in the wrong!*

*So, oft in theologic wars, the disputants, I ween,*

*Rail on in utter ignorance, of what each other mean,*

*And prate about an Elephant*

*Not one of them has seen!*

John Godfried Saxe [1]

The parable of the blind men and the elephant has long illustrated the importance of considering multiple viewpoints in order to see the full picture. The story tells of a group of blind men who are asked to characterize an elephant by touching a different part of its body, such as the tail, the ear, or the trunk. The result is that the elephant is universally mischaracterized because each man makes assumptions based on his partial interaction with the animal. The moral of the parable is that in order to attain a comprehensive version of the truth, we must first acknowledge our limitations and seek out other perspectives.

This parable mirrors conventional healthcare. Current medical practice routinely emphasizes treating the “diagnosis” at the expense of the patient. Doing so results in clinical “blindness” that inhibits best practice and limits comprehensive care.

For this reason, conventional medicine is increasingly trending toward interdisciplinary practice [2,3,4]. This practice acknowledges the patient and the person thereby targeting and treating the whole system.

One way to increase interdisciplinary practice is to promote integrative health care. This involves combining standard medical treatment with mind-body practices. This holistic approach to health care includes treating all parts of the patient, including the “mental, emotional, functional, spiritual, social and community aspects” [5].

Mind and body practices are centered around the patient-practitioner relationship and incorporate all available evidence-based therapies to treat the patient [6]. Acupuncturists, yoga therapists, and creative arts therapists engage in non-traditional mind-body practices that fall under this umbrella. Yoga therapists and creative arts therapists are mental health clinicians trained in psychotherapy and the psychology of their modality (yoga, art, music, dance, and poetry).

At NewYork-Presbyterian Morgan Stanley Children’s Hospital, Columbia University Medical Center, mind-body therapies serve a variety of diverse populations. In an effort to advance integrative health care within this major metropolitan academic institution, these writers embarked upon several hospital-wide initiatives to educate the medical community on evidence-based mind-body interventions. To change culture, they organized a full day conference entitled Creative Arts Therapies Conversations in Healthcare and Therapeutic Transformations (CAT CHATTs): “*What Does Health Look Like in Healthcare Today? Sustaining Identity as an Integrative Therapist in Medicine*”. They invited art, music, dance-movement and yoga therapists, chaplains, physicians, social workers, nurses, psychiatrists, and acupuncturists from major academic institutions to present and discuss numerous ways integrative therapists yield best practice, mitigate effects of invasive procedures, treat physical and emotional pain, and increase communication among staff and with family members for responsible care. This event was supported by NewYork-Presbyterian Morgan Stanley Children’s Hospital (NYP MSCH) and attended by approximately 100 professionals representing all levels of health care providers across New York State. Feedback from evaluations was overwhelmingly favorable and highlighted the need to make integrative healthcare a universal practice in conventional medical settings.

The following are vignettes from five different integrative clinicians at our urban teaching hospital. They serve to illustrate how integrative therapies are effective and evidence-based disciplines that when used in conjunction with standard medical treatment can promote best practice. All patients’ names and identifiers have been changed or replaced with initials to maintain anonymity and privacy.

## 2. Acupuncture in the Pediatric Emergency Department

“Doc, need you in room 23 stat! We have an eight-year-old boy with paraphimosis since last night and clinic just sent him to us for reduction!” It’s now 1:30 p.m. and the foreskin has been in the retracted position since CJ showered last night over 15 h ago. CJ appeared anxious and tearful due to pain from a swollen and red foreskin. After placing him in a Trendelenburg position, topical 2% Lidocaine and D50W plus ice were placed on the area to decrease pain and swelling. Next, intranasal Fentanyl was administered for pain and a child life specialist was at the bedside, providing breathing coaching and an iPad for distraction. The first attempt to release the foreskin was met with such pain and crying that the procedure was aborted. Urology was in the operating room, unavailable to assist. Should I give CJ another dose of opioids? Inject local anesthetics, but risk causing further swelling and emotional trauma? In this case, I offered the family auricular acupuncture to decrease CJ’s pain and anxiety.

Oftentimes, children who are in pain or waiting to undergo a painful procedure are already afraid and anxious. In order to break this tension and dispel their trepidation of acupuncture, I would often jokingly say, “The acupuncture needle is the biggest one in our whole emergency room”! and then dramatically pull out a tiny needle as everyone laughs. To introduce acupuncture to CJ and his family, I first showed them how filamentous the acupuncture needle is by bending it along my finger. Next, I mimicked the pin prick needle sensation by gently pinching the dorsum of his hand while saying, “You might feel the acupuncture needle like a little pinch”. This process helped to put CJ and his family more at ease and they agreed to give acupuncture a try. To further empower CJ at this time when he may feel especially vulnerable, I said, “Let me know if the needle bothers you. We can stop anytime you like”. Using the Battlefield auricular acupuncture protocol [7], CJ calmly allowed me to place five needles in his right auricle. Within minutes we were able to perform a second attempt and successfully reduced the paraphimosis manually, thus avoiding surgery. CJ, his parents, and our emergency medical team were all very happy with the outcome.

This case demonstrates how a child benefited from a collaborative effort using standard pain medications and other non-pharmacologic therapies, including breathing techniques, distraction with iPad, and acupuncture. This multimodal, integrated approach resulted in a successful outcome- something no single therapy could have accomplished.

Acupuncture has been used in China for thousands of years to treat pain [8] and is recognized by the National Institutes of Health as a safe and effective therapy for pain [9]. The mechanisms for acupuncture analgesia have been extensively studied and include the involvement of the autonomic nervous system, endogenous endorphin release, brain region modulation, connective tissue and local needling effects [10,11,12,13]. While many caretakers have heard of acupuncture, most have never heard of its use in pediatrics and are pleasantly surprised when acupuncture is offered in the Pediatric Emergency Department (PED).

Acupuncture is often offered to pediatric patients undergoing radiation and chemotherapy on the Hematology-Oncology service at NYP MSCH because it mitigates symptoms, including nausea, vomiting, pain, and anxiety [14,15,16]. For children with sickle cell pain crisis who present to the MSCH PED, acupuncture is sometimes used in conjunction with standard pharmaceuticals to help decrease pain and anxiety [17], and the amount of opioids needed.

Nursing and physician colleagues in the PED have witnessed first-hand the positive result of acupuncture. For this reason they frequently refer patients for acupuncture when standard of care, including pharmaceuticals, fails to achieve sufficient pain relief. In a study conducted at NYP MSCH PED, patients were treated with acupuncture for diverse painful conditions, including headaches, otitis externa, torticollis, constipation, dysmenorrhea, and knee and ankle sprains. Acupuncture was well accepted, with 96% of patients and families reporting satisfaction with acupuncture and 52% achieving complete pain resolution. Unlike pharmaceuticals, acupuncture offers the additional advantage of low adverse side effects, and avoids medication interactions. Given our nation’s opioid crisis, the integrative practices at NYP MSCH PED conform to the recommendations from the Centers for Disease Control and Prevention and the U.S Food and Drug Administration that nonpharmacologic therapies, including acupuncture, be offered to treat patients with pain [18,19].

It is gratifying to be able to relieve a patient’s pain and suffering; to see a child’s face change from a grimace to a smile. The practice of acupuncture has afforded me opportunities to show families and colleagues that medication alone is not always the solution, nor is it the only answer. With growing evidence on its efficacy and high safety profile, acupuncture may one day become an invaluable staple in our therapeutic armament to help treat pain in pediatrics.

## 3. Pastoral Care as an Integrating Practice

Ravi is a two-and-a-half-year-old boy with a hyphenated Jewish and Italian last name. He has been on the Neurology Unit for one month with seizures. Ravi’s skin is very dark in contrast to everyone else in the room who has very white skin. Ravi is stiff, his limbs are at odd angles, his head is fixed, and he is silent except for occasional moans. Mom and Dad tell me they adopted Ravi after many in vitro attempts and know there is epilepsy in his African-American family of origin. They adore him and Mom talks about how articulate and talented he is. In terms of religious affiliation, Mom is of Jewish heritage and non-practicing. Dad was raised Catholic, but non-practicing, and has for some time sought spiritual expression in Buddhism.

As a chaplain, I am attuned to the different languages and styles people use to describe their values, the ways in which they cope, or do not. I am curious about how this couple navigates their different spiritual languages. These parents are at their wits’ end and I feel like they are leaking out of the boundaries of their bodies. I am a witness to the leaks; and I am a bucket to catch and hold them. Perhaps the collection will be helpful for later reflection for Mom and Dad, but for now I am a bucket brigade with big ears. Over the course of an hour, Mom unfurls a rage aria; they hate the hospital and everyone in it and are furious at how long it is taking for a diagnosis. Next, she laments, revealing she is terrified that their boy will never be their boy again. She cannot bear considering this loss and she declares herself “desperate” for anything that will help, “even Jewish prayer”. We both smile at “even Jewish prayer” as she tells me she never learned much about her Jewish heritage and is not sure what it might offer. I hear her curiosity expressed in the face of her desperation as a desire to connect to something, to claim something that is both a birth right and a tribal right, perhaps as intense as her desire to be a mother. I feel her words of anger and sorrow pierce my own body and consider the options. How to craft a communication from the depths of her soul to…God? She wants to regain her equanimity, but feels she cannot if she is going to continue to advocate effectively for Ravi. A prayer for healing, but for whom? I center the prayer on text written by a 19th century Moroccan rabbi because his origins are as far-flung as the circumstances. Dad is restrained, but cries when we pray in Hebrew and English around Ravi.

Board certified hospital chaplains are responsible for assessing and supporting the emotional, religious, and/or spiritual needs of patients, families, and staff. In addition to supporting these needs, we are trained to listen and to help people access, re-access, or even develop new coping resources regardless of religious or spiritual affiliations. Chaplains help people remember who they are when they are not in the hospital. The conversations can be confessional, investigative, witnessing, and always confidential unless there is potential harm to self or others. Formal research in evidence-based chaplaincy is still in its infancy, however, existing research points to the power of narrative [20] and the value of pastoral care interventions focused on supporting the spiritual needs of primary caregivers of children with life-limiting illnesses [21].

Some weeks later, Ravi has suffered a brain hemorrhage and cardiac arrest, and is in the ICU. He is sedated on a ventilator, his cognitive condition unknown. Over the weeks, Mom and Dad’s spiritual engagement has consisted of meditation with a Buddhist chaplain and a perfunctory wave to the ICU chaplain. When I see them, their eyes are blank and their vitality is sapped. While Ravi has been steadily losing his muscle tone, his parents have begun to lose their person tone. They have so profoundly passed beyond the boundaries of their bodies that they have quietly imploded, and the outlines of who they are in the world as employees, co-workers, friends, and parents are slowly being erased. They tell me they are using meditation to have a moment to “shut down” their thinking and seek a moment of refuge “to not feel”. They are no longer seeking refuge in spiritual resources because they see no purpose. Ravi has become a stranger to them, and they to themselves. They appear as shadows.

Pastoral care in the hospital setting is about identity recalibration. The chaplain looks for the shards of a shattered identity and the ways in which to help reunite them. The intention is to pause the chaos and co-create with the patient, or family, or staff a pathway through the obstacles to find and reclaim a sense of self.

In Ravi’s case, however, there is no clear pathway to reunion, nor definitive resolution. The tragedy of this story is multi-layered. I cannot fix the situation. Instead, I listen to the loss and reflect back the confusion. I hold the myriad of desperate emotions flying around the room and help them to integrate this part of their life story while rebuilding their person tone through reflection of the unwanted emotions they are fiercely trying to shed. I intervene in the moment as each conversation unfolds and I can only hope that my practice in some way helps them to begin to find meaning in the shadows.

## 4. Art Therapy and the Practice of Mirroring Medical Trauma

“Ewww, Alan’s toilet has that thing hanging there with his urine in it, that’s gross”. Alan’s older brother was referring to the urinal. Alan is a tiny 10-year-old boy who speaks in a small inaudible voice, occupying little space in his room, contrary to his older brother and sister. His mother, nervous in nature, and his father, calm yet distant, routinely accompany him to the clinic where he receives treatments for his astrocytoma.

I was consulted to assess for and provide art therapy services throughout Alan’s long and arduous treatment course. Initially he presented with flat affect, withholding of feelings and what appeared to be an absorption of his mother’s anxiety that was present during every medical encounter.

The creative process of art making mirrors life itself. Both process and product serve as a bridge from an individual’s inner world to the outside. Art therapists actively look and listen for imagery, symbols and metaphors, and use them to help explore pre-conscious content with patients. The process of exploring art media, creating and talking about the art offers patients increased opportunities to find more compatible relationships to potentially overwhelming thoughts and feelings [22,23].

Hospitalized children may not have the words to express what they are thinking or feeling, or these words may not do their experience justice. Art, then, serves as a translator to communicate internalized emotions to the important people in the room. Images offer children opportunities to externalize their content safely while the art therapist helps them find personal meaning in their work [24]. Finding meaning, in turn helps to alleviate feelings of anxiety, depression and pain that often accompany children with chronic illnesses [25,26,27].

Alan reported that his G-tube feedings resulted in “excruciating” pain, the primary symptom which led to his current hospitalization. In our first art therapy session, Alan approached the art making inauspiciously, carefully and with unwavering focus. He chose art materials with intention, requesting to work with non-traditional media, and repurposing mundane objects that he altered into a three-dimensional environment.

Typically, children select paints, pastels or clay to form their visual expressions. Alan used a paint brush container as the central sculptural component in his artwork where he used string to attach plastic aquatic figurines from the top allowing them to dangle inside. In art therapy, these two attributes of using non-traditional materials and repurposing objects (e.g., the process of transforming ordinary objects to significantly alter their appearance or use) indicate flexibility, creative problem solving and adaptability.

“That’s not an art material Alan, choose something that is in her [my] basket”. His mother’s reprimands appeared to imply that Alan’s behavior was impolite or defying the rules- in contrast to what I assessed to be a series of creative and resilient solutions. I wondered if this was a mother’s instinct to protect her child and find control in this desperate situation. 

Adhering a plastic transparent circular container (paint brush cleaner) to the center of a green painted circular cardboard base, Alan began to redefine and transform the objects.

Midway through the session, the oncology attending physician humbly entered the room. Alan’s mother stood up to meet him at the foot of the bed, his words stacking like bricks in her arms as he delivered the unresolved news about Alan’s G-tube infection. Upon his exit, Alan’s mother and father murmured their discouragement and concern for Alan’s unexplainable pain.

Shortly after this, the surgical team arrived. “Looks like fun”, a resident commented to Alan about his art. Alan gave no response. Surrounded by the team, his infected G-tube site was examined. I transitioned to the back of the room to be with his siblings who were observing from a distance. “Ewwwww”, exclaimed his sister, “I just saw Alan’s feeding tube site, gross”.

Alan remained quiet, frozen and naked.

As the team discussed their impressions with the parents, Alan continued with his art, selecting a variety of jungle animal figurines and gluing more than twenty tigers, elephants, giraffes and hippos around the perimeter of the circular base, encircling the large ominous container in the center.

“That’s too many animals, save some for the other children”, prompted Alan’s mother once again. Alan’s expressive non-verbal voice is scolded, minimized and criticized as if to say, ‘Don’t take up that much space, stop taking from others and use unforbidden objects. Stay quiet and unseen.’

Unexpectedly, Alan’s roar pierced the room bringing everyone to a halt. “STOP!” He yelled in response to his brother’s persistent attempt to look at his feeding tube site. “Why can’t I see it?” he whined. He silenced the pervasive buzz in the room, “Because I don’t trust you!”

“Whom do you trust?” I asked.

His mother and father stood frozen, holding their son’s eyes with their own. Tears poured down Alan’s cheeks, “I’m scared”. His parents, like bookends, stood on alternate sides of him. “There’s a hole in my side”. Alan’s fear, resounding and exposed, was now unavoidable.

Alan’s art- a depiction of animals surrounding the ‘watering hole’- communicated ‘observation’, ‘vigilance’ and ‘feeding’, all of which were prominent themes from his medical experience. In this moment he was surrounded by teams peering at his feeding tube, his family verbally and visually poking at an area which was the very hole that fed and sustained him. Throughout his exam he exhibited a hypervigilance to incoming danger (i.e., the medical team and his feeds). The container that water is poured into is the focal point in his art piece and a reflection of his own body. Occupying all dimensions of space, the animals’ small stillness reflects the vulnerable unknown. The myriad of feelings elicited from the imagery mirror different aspects of Alan and his experience during this pre-surgical exam and a visit from his physician. Alan’s voicelessness in his treatment was finally unleashed in this compelling moment, providing him an uncensored expression of his fears. Had the surgical team been in the room when Alan was able to clearly verbalize his fears, it’s possible that the team may have listened and responded differently throughout the exam. And alternatively, the response, “Looks like fun” to Alan’s ‘watering hole’ sculpture would not minimize his expression but rather validate it with “Looks like you have a lot to say”.

As an art psychotherapist, I enter into symbolic and metaphoric language to meet the child in his/her inner world. It is humbling to be invited into this space and experience his/her story through imagery and expressive language. The presence of disease in the body can be without symptoms. So too, meaningful messages can exist in a child, and like that of disease, the meaning can be unknown until it is ready to be revealed. In the case of Alan, the unknown became a shared space for Alan, his illness, his art, and me.

## 5. Music Therapy and Interdisciplinary Co-treatments to Creatively Address Complex Medical Needs

“We can beat the bad man, make him go away, if we sing loud enough...All the way up to GOD!” Josiah was a typically developing vivacious seven-year-old boy with a history of prolonged hospitalization and chronic illness awaiting heart transplant. During the first month of his admission, Josiah appeared to exhibit increased signs of anxiety and regression so the interdisciplinary team asked me to assess him for music therapy services. Music therapy as defined by the American Music Therapy Association is “the clinical and evidence-based use of music interventions to accomplish individualized goals within a therapeutic relationship” [28].

In our first session, I quickly established rapport with Josiah through active music making and musical improvisation (e.g., playing extemporized musical rhythms, tempos, dynamics, and melodies composed in the moment without previous preparation on varied rhythmic and harmonic instruments, including drums, guitar, xylophones, and shakers). His initial improvisations presented as playful, melodic, and heavily percussive displaying steady, organized rhythms and lively tempos. His final musical improvisation, however, changed significantly. It became increasingly chaotic, displayed sharp contrasting rhythms in a disorganized pattern and was punctuated by strong accentuated beats. Mid-way into this improvisation Josiah stopped abruptly and pointed directly to a Halloween face mask adjacent to him on the bed. He placed his finger over his mouth and with wide-eyed affect whispered, “we need to be very quiet... so we don’t wake the bad man”. He then proceeded to sing softly in an effort to avoid being captured by this “bad man” monster who wanted to “silence” his music.

As a music psychotherapist, I use my modality to help patients explore, express, and discover their internal worlds by inviting ongoing musical and verbal dialogues between their conscious and unconscious selves [29]. During our first session, I assessed that Josiah’s impromptu musical dialogue between his conscious self and a fictional “bad man” monster may have been prompted by a need to safely and creatively explore, express, and process difficult thoughts and feelings surrounding his chronic illness, prolonged hospitalization, isolation, and family separation. What was most interesting to me during our first encounter was that in calling out to a higher power at the conclusion of the session, Josiah appeared to achieve some kind of resolution. By singing “loudly, all the way up to God” he had triumphantly silenced the “bad man”, holding him captive. Essentially, Josiah had discovered comfort, personal strength, power, and self-agency by engaging in musical prayer.

Pre-transplant, my clinical work with Josiah was predominantly psychodynamic in that I continued to invite him to engage in musical dialogues between his conscious and unconscious selves. In these ongoing dialogues, he explored a variety of recurring themes in the music, including ‘silence/sound’, ‘dominance/submission’, ‘danger/safety’, ‘fear/courage’, ‘separation/reunion’, ‘helplessness/empowerment’, and ‘salvation’.

Mid-way through his admission, Josiah was successfully transplanted, however, postoperative complications led to a cardiac arrest that resulted in anoxic brain injury and substantial decompensation. Following this event, the family was in crisis and the team reported uncertainty as to Josiah’s neurological outcome.

For me, the shift was just as dramatic. In many ways, Josiah’s musical journey paralleled his cardiac journey; his music had stopped abruptly, unexpectedly, and without warning, leaving in its wake a painfully uncomfortable silence. Clinical music therapists are trained to create space in the music for our patients because rests are just as important as the notes. But how could I begin to honor Josiah’s notes when his voice as I knew it had been silenced?

As a result of this experience, my clinical practice evolved to become more flexible, innovative, and creative. I began by increasing communication with team members to help identify common goals and innovative solutions aimed at addressing a variety of barriers to care. Josiah displayed extreme spasticity in his lower extremities, which created limited mobility, and he had difficulty maintaining autonomic stability. This was most evident during physical and occupational therapy sessions when he often became tachycardic and tachypneic. In these sessions, he also displayed visible and audible signs of distress and emotional dysregulation, including moaning, crying, contracted limbs, and a constricted, tearful affect.

In response to these barriers, I worked closely with the team to design individualized music therapy co-treatments. To encourage homeostasis during rehabilitation therapy sessions, I played improvised harmonies on the guitar, which emphasized regular, steady rhythms and slow tempos while singing improvised melodies with prolonged phrases or musical meter. This intervention known as *rhythmic entrainment* (a term used to describe the temporal locking process phenomenon in which two or more independent rhythmic processes synchronize with each other) [30] helped to stabilize Josiah’s heart rate and respiration throughout sessions. Entrainment also proved a useful assessment tool for the team. The occupational therapist reported that she was able to more accurately assess his neurological capacity as a result of the musical accompaniment I provided that both activated and relaxed Josiah throughout our session. Because Josiah entrained easily to live music, the speech and language pathologist informed me that live music helped keep Josiah calm, which enabled her to successfully complete a visual, audial, and cognitive assessment to determine his altered baseline. 

In physical therapy sessions, I used a sound and vibration intervention known as *vibroacoustic therapy* [31,32] to minimize pain and increase his limited range of motion. By placing a bass tuned buffalo drum near Josiah’s lower extremities I was able to reduce his hypertonicity and spasticity [33]. Suddenly, the physical therapist was able to increase the passive range of motion and encourage active range of motion when Josiah was able to successfully reach for, grasp, hold, and shake a small bell in time to the music. As a result of increased integrative practice that targeted and treated both psychosocial and physiological symptoms of distress, Josiah’s autonomic stability and emotional regulation slowly improved over time.

A final collaboration with pastoral care led to a co-treatment that provided Josiah’s family the opportunity to give voice to overwhelming feelings of fear and anxiety. I played simple chord intervals (e.g., two sounds differing in pitch) on the guitar and sang an improvised melody to create a musical environment that was secure, contained, and predictable while the chaplain led the family in prayer. This technique known as *vocal holding* consists of the intentional use of playing two alternating chords on a harmonic instrument in combination with improvised singing in order to create a consistent and stable environment [34]. Initially, Josiah’s mother expressed doubt and confusion surrounding the event, “Why would God let this happen?” Throughout the music her demeanor shifted. Toward the end of the session she presented as calm and hopeful, reporting, “I can’t be upset at God… God has a plan. Even though it’s bad, and it’s dark ...We going to get through this. God’s going to pull us through.” In a way we had come full circle; once again, Josiah and his family were seeking comfort, empowerment, and strength in musical prayer.

At this point in the session, Josiah, who had been sleeping in a bed adjacent to our prayer circle, opened his eyes and his mother moved bedside to take his hand. Softly, she began to sing his favorite song, “Oh, the itsy, bitsy spider, went up the water spout...” I quietly fingered chords on my guitar to accompany her as she continued to sing, “Down came the rain, and washed the spider out...” Slowly, family members gathered around the bed holding hands, encircling Josiah as he held her gaze. “Out came the sun, and dried up all the rain...” Josiah opened his mouth slowly, hesitantly groping as his mother sang the last line, “and the itsy, bitsy spider went up the spout ah…” Josiah completed the song, penetrating the silence with his voice at last, “...gin”.

My favorite thing about music therapy is that it is inherently collaborative. Unlike conversation, music happens concurrently offering participants an “I” and “Thou” [35] experience through “We” engagement [36]. ‘We’ engagement, however, can sometimes limit opportunities to unite ‘us’ in clinical practice. By expanding our practice to include multiple voices, we participate in a richer, more dynamic symphony. Ultimately, an integrated practice promotes true harmony in healthcare.

## 6. Yoga Therapy and Meditation: An Integrative Partnership with Patients and Families

The chemotherapy charge nurse texted, “Please come to the Infusion Center to meet a new patient who wants to talk to you about yoga therapy and meditation during her cancer treatment”.

The patient, Stacey, had recently celebrated her 18th birthday. Soon after, she had felt a lump near her right armpit. The staggering diagnosis of stage IV breast cancer with metastases to the liver and spine literally took her breath away. Today was the first of an anticipated 180-day chemotherapy treatment plan to be delivered once every three weeks.

As I approached the infusion area, I paused to center myself so I could be fully present with Stacey—allowing her to be heard, to be seen; holding her in compassionate, loving space. Elizabeth Kubler-Ross said, “Telling your story often and in detail is primal to the grieving process. You must get it out. Grief must be witnessed to be healed [37] (p. 63)”.

I washed my hands, focusing on my own breath: “Take three deep belly breaths, relax into the exhalation, allow your awareness to rest on the present moment”. I continued to practice this deep breathing, and soon Stacey mirrored the gentle rise and fall of my abdomen. Her breathing slowed as I guided her through a 15-min meditation. She said she felt calmer and I saw on the monitor her systolic blood pressure had dropped 15 points. I showed her various breathing techniques to practice at home and during treatments to help manage anxiety and discomfort, and we began to create a plan for a daily meditation practice that was based on her personal preferences.

As a Yoga Therapist in Integrative Oncology, I work closely with Psycho-Oncology and Palliative Care. The cornerstone of our team’s integrative approach is to partner with patients and families, providing attentive, empathic clinical care to address the needs of mind, body, and spirit. I was the first of our team to meet Stacey and immediately suggested she consult our Psychologist and Palliative Nurse Practitioner for help managing anxiety and prospective chemotherapy induced peripheral neuropathy. During her half-year of treatment, our team met regularly to discuss her case, and I supported my colleagues’ behavioral and pharmaceutical interventions by customizing weekly sessions of conscious breathing, meditation, and mindful movement to address her evolving physical and emotional needs.

Mind-body interventions are important self-regulation practices that support psychosocial therapies by fostering general mental well-being, calmness, clarity, and concentration [38,39,40]. They promote sleep, and can ease anxiety and depression [41,42]. Mindfulness meditation, in particular, helps manage anxiety and improve quality of life [43]. It also helps patients reframe their relationship with pain by teaching them to rest in the present moment and observe sensations and thoughts without judgment. Jon Kabat-Zinn, the founder of Mindfulness Based Stress Reduction (MBSR), wrote, “It’s possible to befriend your pain or your fear...rather than feeling that you can’t get anywhere until this thing that bothers you is cut out or walled off or shut down” [44].

There is substantial evidence to support the efficacy of mind-body therapies in the outpatient clinical setting. At its June 2018 annual conference, the American Society of Clinical Oncology (ASCO) endorsed yoga and meditation as adjuvant therapy during conventional breast cancer treatment and survivorship to mitigate the sequelae of acute and late treatment side effects, including anxiety, mood disorder, depression, and to improve quality of life [45].

At the end of her treatment Stacey was declared with *no evidence of disease* and told us that our integrative care had “saved her life” by teaching her how to manage her fear and anxiety, and to help her keep physically active during her difficult treatment. In her mother’s words, “Stacey’s medical oncologists may have eradicated the disease and put her in remission, but her integrative support team gave her back a life worth living”.

It is imperative that we, as integrative therapists, spearhead the effort to create a paradigm shift in the way our healthcare system treats our patients and their loved ones. Mind-body therapies are evidence-based, drug free, cost effective, and available to everyone regardless of cultural background and socioeconomic status. Let us listen closely to our patients and their families, see the elephant in its entirety, and commit to treating the individual, not the disease.

## 7. Results: Barriers to Integrative Therapy Practice

The above vignettes clearly illustrate how integrative therapies address the mental, emotional, functional, spiritual, social, and community domains of the patient, thereby improving the overall patient experience while supporting standard medical treatment outcomes. Despite the overwhelming evidence that points to the effectiveness of integrative healthcare, these writers encountered areas for further consideration.

### 7.1. Funding

Currently, in conventional medical settings, integrative therapy positions are funded in a variety of ways. While many creative arts therapy positions are typically donor funded, at this institution, art and music therapy and pastoral care positions are billed to the cost of room and board. Yoga therapy is funded by a private donor, and acupuncture services in the pediatric emergency department are currently not separately reimbursed. It remains clear that additional cost analysis research is needed to find sustainable solutions to increased integrative programming.

### 7.2. Misses in Appropriation, Placement, Classification, and Understanding

#### 7.2.1. Misappropriation

In conventional medical settings, mind-body practices are often misplaced, misunderstood, and misclassified. This leads to a misappropriation of services, impaired practice and, ultimately, identity confusion regarding the role integrative therapists play within the treatment team. For this reason, clarifying the scope of practice and managing staff and family expectations is paramount.

#### 7.2.2. Misplacement/Misclassification

Misplacement of integrative therapists creates confusion within the interdisciplinary team. Therapists are difficult to locate, often possessing job titles or embedded in departments unrelated to their discipline. For example, a pain specialist may have a hard time locating a mind-body therapist when he/she is identified on his/her identification badge as a recreational therapist and managed by a child life department.

#### 7.2.3. Misunderstanding

Integrative therapists are trained to facilitate and process the expression of anxiety and fear that accompany medical encounters that are interwoven with pain symptomatology [46,47,48,49]. Not all healthcare providers understand the concrete benefits of mind-body interventions and may minimize the complementary benefits integrative therapies can provide. A better understanding of mind-body practices is necessary in order to achieve appropriate integration and promote a more holistic approach to care.

## 8. Discussion: Recommendations for Future Practice

Despite the aforementioned barriers, these writers were able to identify a number of innovative solutions to minimize siloed practice and help break down existing barriers. Through workshops, targeted in-services, interdisciplinary rounding, co-treatments, lectures, grand rounds, and hospital-wide educational forums, including conferences and workshops, medical professionals are provided with opportunities to learn and experience firsthand the benefits of integrative therapies, including art, music, yoga, pastoral care, and acupuncture.

### Innovative Solutions to Promote Increased Integrative Practice

The advancement of integrative health care in a major academic hospital takes time and strategy to promote complementary practice. Below are suggested actions steps to help increase visibility, awareness, and accessibility to integrative services. The recommendations listed are from these writers’ personal accounts and experiences of successful implementation of hospital-wide programs and initiatives.

- Create a customized website with a dedicated email address to promote understanding and classification of integrative therapies.
- Design and distribute educational brochures and marketing materials to staff detailing services and appropriate referral criteria.
- Facilitate weekly in-services for interdisciplinary staff, fellows, residents, medical students, and physicians that lead to better integration of mind-body services in treatment plans. This practice also generates potential funding for additional positions, including expanding services to outpatient or satellite clinics.
- Obtain funding through grant support from established national organizations, such as the American Academy of Pediatrics, Virginia Apgar Academy, American Art Therapy Association, private donors, and foundations to support educational workshops and research.
- Conduct workshops, lectures, and grand rounds for nurses, students, residents, and faculty members, thereby gaining more acceptance and understanding from providers across the hospital on integrative therapists’ role in medicine.
- Publish and present locally, nationally, and internationally on integrative therapy research and practices.
- Create an integrative rotation elective as part of residency training to educate and promote utilization of complementary practices located throughout the hospital.
- Involve integrative therapy students in the creation and co-facilitation of expressive therapy pilot programs for outpatient clinics, which increases complementary services to more patients and families in need.

## 9. Conclusions

A cultural shift in this conventional medical setting is growing as a result of increased visibility, education, understanding, and collaboration, but the need for increased integration of mind-body practices still remains. Barriers to true integrative health care include limited funding, misappropriation of services, misclassification, misunderstanding, and continued siloed practice. To affect global change, it is incumbent for all mind-body clinicians in conventional medical settings to join this cultural revolution. This publication is just the first step. A restructuring of conventional medical frameworks is necessary in order to see and treat the whole patient. From revolutionary paradigm shifts and innovative practices to eradicating siloed services, integrative, complementary “wholistic” health is the future.

Organizations that begin to successfully integrate humanistic medicine with evidence-based mind-body practices empower patients to become active partners in their treatment. Such integrative practice reduces harm, sustains ethical and legal professional standards, gives purposeful direction to therapeutic interventions, and enhances the efficacy of conventional treatment plans. It is the future of good medicine. This is the elephant that we can all see and heal.
